# Biotransformation of rare earth oxide nanoparticles eliciting microbiota imbalance

**DOI:** 10.1186/s12989-021-00410-5

**Published:** 2021-04-26

**Authors:** Huizhen Zheng, Zonglin Gu, Yanxia Pan, Jie Chen, Qianqian Xie, Shujuan Xu, Meng Gao, Xiaoming Cai, Shengtang Liu, Weili Wang, Wei Li, Xi Liu, Zaixing Yang, Ruhong Zhou, Ruibin Li

**Affiliations:** 1 State Key Laboratory of Radiation Medicine and Protection, School for Radiological and Interdisciplinary Sciences (RAD-X), Collaborative Innovation Center of Radiological Medicine of Jiangsu Higher Education Institutions, Soochow University, Suzhou, 215123 Jiangsu China; 2 Institute of Quantitative Biology, Department of Physics, Zhejiang University, Hangzhou, 310027 Zhejiang China; 3 School of Public Health, Jiangsu Key Laboratory of Preventive and Translational Medicine for Geriatric Diseases, Soochow University, Suzhou, 215123 Jiangsu China; 4 Department of Chemistry, Columbia University, New York, NY 10027 USA

**Keywords:** Rare earth oxide, Nanotoxicity, Biotransformation, Microbiota imbalance, Pulmonary inflammation

## Abstract

**Background:**

Disruption of microbiota balance may result in severe diseases in animals and phytotoxicity in plants. While substantial concerns have been raised on engineered nanomaterial (ENM) induced hazard effects (e.g.*,* lung inflammation), exploration of the impacts of ENMs on microbiota balance holds great implications.

**Results:**

This study found that rare earth oxide nanoparticles (REOs) among 19 ENMs showed severe toxicity in Gram-negative (G^−^) bacteria, but negligible effects in Gram-positive (G^+^) bacteria. This distinct cytotoxicity was disclosed to associate with the different molecular initiating events of REOs in G^−^ and G^+^ strains. La_2_O_3_ as a representative REOs was demonstrated to transform into LaPO_4_ on G^−^ cell membranes and induce 8.3% dephosphorylation of phospholipids. Molecular dynamics simulations revealed the dephosphorylation induced more than 2-fold increments of phospholipid diffusion constant and an unordered configuration in membranes, eliciting the increments of membrane fluidity and permeability. Notably, the ratios of G^−^/G^+^ reduced from 1.56 to 1.10 in bronchoalveolar lavage fluid from the mice with La_2_O_3_ exposure. Finally, we demonstrated that both IL-6 and neutrophil cells showed strong correlations with G^−^/G^+^ ratios, evidenced by their correlation coefficients with 0.83 and 0.92, respectively.

**Conclusions:**

This study deciphered the distinct toxic mechanisms of La_2_O_3_ as a representative REO in G^−^ and G^+^ bacteria and disclosed that La_2_O_3_-induced membrane damages of G^−^ cells cumulated into pulmonary microbiota imbalance exhibiting synergistic pulmonary toxicity. Overall, these findings offered new insights to understand the hazard effects induced by REOs.

**Supplementary Information:**

The online version contains supplementary material available at 10.1186/s12989-021-00410-5.

## Background

Microorganisms play critical roles in animal metabolism and Earth’s biogeochemical cycles as they are responsible for litter decomposition (e.g.*, Bacillus subtilis* and *Enterobacter*), nitrogen fixation (like *Rhizobium*), oxygen production (e.g.*, Synechococcus* and *Cyanobacteria*), as well as nutrition (e.g.*, Lactobacillus* and *Bifidobacterium*) and energy (e.g.*, Shewanella*) supplies [1, 2]. After several billion years of evolution, versatile microbes have evolved diverse local communities [3], a.k.a. microbiota, to maintain mutualistic relationships with humans, animals and plants. Any disruption of microbial community may elicit severe diseases in living systems or spell disasters for local ecosystems [4]. Remarkably, certain pollutants generated from industrial developments have been demonstrated to significantly affect microbial communities, resulting in severe environmental and health issues. For instance, the exposure of environmental persistent organic pollutants (POPs), e.g.*,* 2,3,7,8-tetrachlorodibenzofuran, with 24 μg/kg in the diet for 5 days may altered the gut microbiota by shifting the ratio of Firmicutes to Bacteroidetes, which was associated with the alteration of bile acid metabolism [5]. Moreover, imbalance of microbial community in lung may have an important role in progression of pulmonary disorders, such as chronic obstructive pulmonary disease, asthma, cystic fibrosis and lung cancer [6]. Since inhalable pollutants are the causes of lung diseases, a focus shift towards the study of their toxicological effects on microbial community has come into questions.

During the past decade, a number of nanoparticles have potential environmental exposure risk during their lifecycles including material synthesis and transportation, nanoproduct fabrication, consumption and recycle [7]. Some engineered nanomaterials (ENMs) have been found to affect the biological function and viability of microorganisms [8]. For instance, GO and MoS_2_ nanomaterials with mechanical damage and surface oxidative activities were reported to kill bacteria by disrupting cellular membranes [9, 10]. Vanadium pentoxide nanoparticles and Pt hollow nanomaterials with peroxidase-like activities were demonstrated to impair the activities of both Gram-negative (G^−^) and Gram-positive (G^+^) bacteria [11, 12]. In general, these nanomaterials have been reported to show non-preference for diverse microorganisms, exhibiting indiscriminate toxicities via ROS generation [10], membrane damage [13], DNA duplication inhibition [14], etc. However, it is unclear whether any ENM may affect microbiota balance.

In this study, we investigated the impacts of 19 ENMs on G^−^ and G^+^ bacteria and specifically focused on the distinct toxicity effects of rare earth oxide (REO) nanoparticles in G^+^ and G^−^ bacteria. To decipher the molecular initiating events (MIEs) of the hazard effect, we visualized the biotransformation of La_2_O_3_ at nano-bio interfaces and revealed the dynamical interaction process by molecular dynamics (MD) simulations. Based on the MD simulation predictions, we then performed fluorescence polarization and β-galactosidase release assays to validate the impacts of La_2_O_3_ on membrane fluidity and permeability, respectively. The effects of La_2_O_3_ were validated in lung and soil microbial communities by Gram staining and 16S rRNA-based sequencing approaches. We examined the impacts of microflora imbalance on lung tissues and made a hypothesis that the biotransformation of La_2_O_3_ in lung may elicit the microbiota imbalance and pulmonary toxicity.

## Methods

### Materials

Bacterial culture medium including Luria-Bertani (LB) broth and Tryptic Soy Broth (TSB) were purchased from Sangon Biotech (Shanghai, China). The sources of ENMs, including REOs and other nanoparticles were displayed in Table S1. 1,2-distearoyl-sn-glycero-3-phosphocholine (PC, 18:0/18:0, Mw: 790, No. 850365) was purchased from Avanti (USA). Beta-Glo Assay System (E4720) used for membrane permeability test was purchased from Promega (USA). 4′-(trimethylammonio) diphenylhexatriene (AM9625) was obtained from AAT Bioquest Inc. (USA) to determine the membrane fluidity. Bacterial counting colorimetric assay kit (K511) used to quantify the viability of bacteria was purchased from Biovision Inc. (USA). Live/Dead BacLight bacterial viability kit (L7007) from Thermo Fisher (USA) was used to examine the viability of bacterial populations by a fluorescence microscope. Alexa Fluor® 594 conjugate of wheat germ agglutinin (WGA, W11262) and 4′,6-Diamidino-2-Phenylindole (DAPI, D3571) purchased from Thermo Fisher (USA) were used to examined G^+^ and G^−^ bacteria. Colistin (C4461) was purchased from Sigma-Aldrich (St. Louis, Mo, USA). ELISA kits for detection of IL-1β and IL-6 were purchased from BD biosciences (San Jose, CA, USA); MCP-1 ELISA kit was purchased from R&D (Minneapolis, MN, USA). Quick-Diff staining kit was purchased from Yeasen Biotechnology (Shanghai, China).

### Transmission electron microscope (TEM) and X-ray diffraction (XRD) characterizations

La_2_O_3_ or LaPO_4_ (5 mg/mL) was diluted into alcohol at 50 μg/mL and dispersed by a probe sonicator (Scientz-IID, Scientz, China) at 32 W for 30 s (10 s on, 2 s off). Drops of alcohol suspensions were placed on copper grids for air drying at room temperature. Then they were observed by TEM at 120 kV (Tecnai G20, FEI, USA). After the nanoparticles drying at vacuum desiccation for overnight, their crystal structures were detected by XRD (D8 Advance, Bruker, Germany) with Cu Kα radiation.

*E. coli* cells (~ 10^8^ CFUs) at exponential phase were exposed to 1 mL saline solution containing 250 μg/mL La_2_O_3_ in a 1.5 mL Eppendorf tube. After 2 h incubation in a shaker (200 rpm, 37 °C), the treated cells were collected by 6000 g centrifugation for 5 min and fixed in 2.5% glutaraldehyde for 30 min at room temperature. Followed by overnight incubation, osmium tetroxide (1% w/v) was used to stain the fixed cells for 1 h. Then we dehydrated the samples in a graded series of ethanol (30, 50, 70, 90, 100%). The samples were finally embedded in Epon, followed by propylene oxide treatment. Cell sections were cut by an ultramicrotome (EM UC7, Leica, Wetzlar, Germany), picked up on Formvar-coated copper grids for further staining by uranyl acetate and Reynolds lead citrate. The grids were observed by TEM (Tecnai G2 spirit BioTwin, FEI, USA) coupled with energy-dispersive X-ray (EDX).

### Bacterial activity assessment

*E. coli* cells were cultured at LB broth, and *B. subtilis* cells were cultured in medium containing 5.0 g peptone, 3.0 g beef extract and 5.0 g NaCl per liter. When they were grown to exponential phase (OD_600_ at 0.6 to 0.8), the cells were collected by centrifugation of 1 mL suspensions at 6000 g for 5 min and dispersed in 5 mL saline. ENM suspensions were prepared by dilution of 5 mg/mL ENM stock solutions into saline solutions. After dispersion by a probe sonicator (Scientz-IID, Scientz, China) at 32 W for 15 s, gradient concentrations (0, 62.5, 125, 250 and 500 μg/mL) of ENM suspension were acquired and added into 96-well plates (100 μL/well). Aliquots of 100 μL bacterial suspension were exposed to 100 μL ENMs in 96-well plates. The final concentrations of ENMs were 0, 31.75, 62.5, 125 and 250 μg/mL. After 2 h incubation at 37 °C in a shaker (200 rpm) away from light, the bacterial activities were determined by a bacterial counting colorimetric assay kit according to the manufacture’s protocol. In detail, aliquots of 20 μL colorimetric assay solutions were added into each well for additional 30–60 min incubation. Three replicates were performed at each dosage. We collected the supernatants by centrifugation at 1000 g for 5 min (Sorvall ST16R, Thermo, USA) and transferred them into a new 96-well plate for OD detection at 460 nm by a microplate reader (Synergy NEO HTS, Biotek, USA). Cell viabilities were calculated by formula 1:

1 $$ \mathrm{Cell}\ \mathrm{viability}\ \left(\%\right)=\frac{{\mathrm{OD}}_{\mathrm{x}}-{\mathrm{OD}}_{\mathrm{b}}}{{\mathrm{OD}}_0-{\mathrm{OD}}_{\mathrm{b}}}\times 100\% $$

where OD_x_, OD_0_ and OD_b_ are the absorbance of ENM-treated bacteria, untreated bacteria and medium blanks, respectively.

### Confocal microscopy imaging of bacterial cells

*E.coli* and *B. subtilis* cells (~ 10^8^ CFUs) cultured at exponential phase were collected and washed twice by saline solution. Then the bacterial cells were centrifuged at 6000 g for 5 min (Allegra 64R, Beckman, USA) and dispersed in 0.5 mL saline containing 250 μg/mL La_2_O_3_ nanoparticles. After 2 h incubation, aliquots of 0.5 mL bacterial suspension were stained for 15–20 min at room temperature by mixing 1.5 μL Live/Dead bacterial viability kit. Aliquots of 5 μL stained cells were subsequently dropped on a microslide and visualized by a confocal microscope (UltraView VoX, PerkinElmer, USA) with a 100X oil immersion objective at 488/500 nm for SYTO 9 and 488/635 nm for prodium iodide (PI). WGA conjugated with Alexa Fluor 594 was used to differentiate G^+^ strains. The microbial communities collected from soil or animal lungs were suspended in 1 mL saline solutions containing 1 μg/mL WGA and 10 μg/mL DAPI. After 15 min incubation at room temperature, cells were collected by centrifugation at 6000 g for 5 min and sufficiently washed by saline for three times. Then the bacterial cells were dispersed in 100 μL saline and visualized by a confocal microscope (FV1200, Olympus, Japan) with a 60X oil immersion objective at excitation/emission wavelengths of 405/450 nm for blue fluorescence and 488/635 nm for red fluorescence. Six images were obtained for each sample.

### Colony forming unit (CFU) counting

CFU assay was employed to determine the toxicity of La_2_O_3_, TiO_2_ and Ag nanoparticles in *E.coli*, *P. aeruginosa*, *S. aureus* and *B. subtilis.* In detail, *E.coli* and *P. aeruginosa* were cultured in LB broth. *S. aureus* was cultured in TSB broth, and *B. subtilis* was cultured in its medium containing 5.0 g peptone, 3.0 g beef extract and 5.0 g NaCl per liter. Cell suspensions (1 mL) at exponential phase were centrifuged at 6000 g for 5 min (Allegra 64R, Beckman, USA). After washing twice by saline, the cell pellets were dispersed in 1 mL saline solutions containing 250 μg/mL nanoparticles. After 2 h incubation, seven serial dilutions were performed by adding 1 mL of bacterial suspension into 9 mL saline. Aliquots of 100 μL diluted bacterial suspension were added to agar plates with 10 cm diameter. After 24 h incubation at 37 °C, appropriate colonies (20 ~ 100) on agar plates were selected to count colony numbers. Three replicates were performed at each dilution. CFU and survival rates were calculated by formula 2 and 3:

2 $$ \mathrm{CFU}\ \left({\mathrm{mL}}^{-1}\right)={\mathrm{C}}_{\mathrm{n}}/0.1\times {10}^{\mathrm{n}} $$

3 $$ \mathrm{Survival}\ \mathrm{rate}\ \left(\%\right)=\left({\mathrm{CFU}}_{\mathrm{ENM}}/\mathrm{CFU}\right)\times 100\% $$

Where C_n_ is the colony number on agar plates at n^th^ dilution; CFU_ENM_ and CFU represent the colony numbers of ENM-treated and untreated bacteria, respectively.

### La_2_O_3_-lipid interaction measurement

In order to investigate the detailed interaction between nanoparticle and cell membrane, phosphatidylcholine (PC) liposomes were prepared to mimic the cell membrane by a film dispersion method. In detail, we dissolved 2 mg PC in 2 mL of chloroform and transferred them into a 10 mL round-bottom flask. A lipid film was prepared by removing chloroform with a rotary evaporator at 160 mbar, 150 rpm for 30 min. After that, ultrasonic dispersion was performed in 2 mL DI H_2_O for 30 min. PC liposomes were collected by centrifugation at 30000 g for 5 min and re-suspended in 2 mL saline. La_2_O_3_ (250 μg/mL) was exposed to 1 mL liposome solutions in a 2 mL Eppendorf tube. After 4 h incubation in a shaker, the pellets were obtained by centrifugation at 30000 g for 5 min and fully suspended in 1 mL chloroform by 5 min vortex for extraction of PC molecules. The suspensions were centrifuged at 15000 g for 5 min to collect the supernatants, which were subjected to liquid chromatography coupled to mass spectrometry (LC-MS) analysis according to a reported protocol [15]. In detail, it was conducted on an Agilent 1100 Series LC system (Agilent Technologies, Santa Clara, CA, USA) with a reversed phase HPLC column (2.1 mm × 150 mm, 100 Å, 5 μm) coupled to a mass spectrometer (Thermo Fisher Scientific, Waltham, MA, USA). Samples were separated with an isocratic gradient of isopropanol and acetonitrile (45:55, v/v) containing 10 mM ammounium formate at 200 μL/min flow rate. Full scan MS detection was conducted in positive ionization mode to collect 100 to 1000 m/z data, which was analyzed by Xcalibur software.

### Molecular dynamics simulation

Coarse-grained model [16] was employed to simulate the bacterial membrane by using CHARMM-GUI Martini Bilayer Maker [17, 18] with the outer membrane modeled by pure POPE and the inner membrane by a mixture of POPE and POPG (3,1 ratio), similar to our previous protocol [13]. The initial membrane had a lateral dimension of 20 × 20 nm. Two simulation systems were constructed, with one for the control system (i.e. the normal bacterial membrane) and the other for the dephosphorylated system. In the dephosphorylated system, some POPE lipids (proportion of ~ 9.0% based on LC-MS quantification) located on the outer leaflet were “mutated” to dephosphorylated POPE (de-POPE) by deleting NH3 and PO4 beads to mimic our experimental settings. The MD simulations were performed using GROMACS software package (version 5.0.2) [19] and the Martini force field (version 2.2) [20]. The temperature and pressure were controlled by velocity-rescaling thermostat (at 303 K) [21] and the Parrinello-Rahman barostat (1 bar semi-isotropic pressure coupling) [22], respectively. The periodic boundary conditions (PBC) were implemented in all directions (X, Y and Z). We performed all simulations with an integration time step of 20 fs. Electrostatic interactions were modulated with the reaction field method using dielectric constants of 15 and infinity for charge screening in the short-range and long-range regimes, following the recommended simulation settings for coarse-grained Martini (version 2). The short-range cutoff distance for the electrostatic interactions was set to 1.2 nm. The potential shift Verlet scheme was used to cut off the Lennard-Jones potential at long ranges. All simulations followed the basic protocol obtained from CHARMM-GUI. Each system ran for 1 μs (1000 ns). The order parameter (−*S*_CD_) of coarse-grained lipids could be calculated based on the equation following previous studies [23], which reflected the quantification of the relative order of the hydrocarbon tails.

4 $$ {S}_{\mathrm{CD}}=\frac{1}{2}\left(3{\cos}^2{\theta}_i-1\right) $$

*θ*_*i*_ is the angle between the bilayer normal and the local orientation of neighboring hydrocarbon beads of the lipids.

### Membrane fluidity and permeability assays

*E. coli* and *B. subtilis* cells at exponential phase were collected by centrifugation at 6000 g for 5 min and suspended in 1 mL saline solutions, respectively. La_2_O_3_ or LaPO_4_ nanoparticles at 250 μg/mL were exposed to 500 μL bacterial suspensions in 1.5 mL Eppendorf tubes. After 2 h incubation at 37 °C in a shaker, the suspensions were mixed with 5 μL TMA-DPH stock solutions (2 mM) and incubated for 30 min at 37 °C. The incubated cells were collected by centrifugation at 6000 g for 5 min and dispersed in 500 μL saline solution after washing three times. Aliquots of 100 μL bacterial suspensions were transferred into a 96-well plate and examined at ex/em 360/460 nm by a microplate reader (Synergy NEO HTS, Biotek, USA) with a 66 FP module. Three replicates were performed for each sample. The obtained fluorescence polarization (FP) value of TMA-DPH is a reliable index for membrane fluidity evaluation [24].

A β-Glo assay reagent (Promega) was used to detect β-galactosidase release from damaged cells for membrane permeability evaluation. In detail, *E. coli* and *B. subtilis* cells (~ 10^8^ CFUs) were incubated with 500 μL saline solutions containing 250 μg/mL nanoparticles (La_2_O_3_ or LaPO_4_) or 500 μL cell lysis buffer (positive control). The untreated cells were used as negative control. After 2 h incubation at 37 °C in a shaker, the cells were centrifuged at 15000 g for 5 min to collect supernatants. Aliquots of 50 μL supernatants were transferred into 96-well plates to react with 50 μL β-galactosidase substrate for 30 min at room temperature. The luminescence intensities were determined by a microplate reader (Synergy NEO HTS, Biotek, USA). Three replicates were performed for each sample. The cell membrane permeability was calculated by following formula 5:

5 $$ \upbeta \hbox{-} \mathrm{galactosidase}\kern0.5em \mathrm{release}\kern0.5em \left(\%\right)=\frac{I_{\mathrm{a}}}{I_{\mathrm{neg}}+{I}_{\mathrm{pos}}}\times 100\% $$

Where *I*_a_, *I*_neg_ and *I*_pos_ represent the luminescence intensities of samples, negative and positive controls, respectively.

### Animal experiments

Eight-week-old male C57BL/6 mice from Peng Sheng Biological Technology (Nanjing, Jiangsu, China) were housed in groups of two to four mice per cage under standard laboratory conditions (25 °C; 60% relative humidity and 12 h light/12 h dark cycle) by Soochow University guidelines. Our animal protocols were approved by the Committee of Animal Research and Ethics in Soochow University. Ten mice were anesthetized by intraperitoneal injection of sodium pentobarbital (200 mg/Kg) in a total volume of 80–100 μL and randomly divided into two groups. Each group included five mice. One group was exposed to 2 mg/Kg La_2_O_3_ by oropharyngeal aspiration. In detail, aliquots of 50 μL nanoparticle suspensions were instilled at the back of the tongue in anesthetized animals (*n* = 5). Another group subjected to 50 μL saline solutions was used as control. After 24 h exposure, over-anesthetized mice (400 mg/Kg) were sacrificed to collect BALF by bilateral thoracotomy. Furthermore, to explore the relationships between lung inflammation and the microflora imbalance, mice were firstly exposed to 2 mg/Kg La_2_O_3_ as previous description. At 4 h post injection, colistin solutions were instilled into mouse lungs (*n* = 5) at different doses (0, 0.6, 2, 4 mg/Kg). Mice merely exposed to 4 mg/Kg colistin were used as a control group. The BALF was obtained by using a 1 mL syringe with a G22 × 1″flat needle after 24 h exposure, which was collected into 2 mL tubes by thrice syringing 0.8 mL PBS to wash airways in animal lungs. While the immune cells in BALF were collected by centrifugation at 800 g for cell counting, the lung microbial communities were obtained by centrifugation at 6000 g for Gram staining. The supernatants were used for detection of IL-6, IL-1β, and MCP-1 by ELISA according to the manufacture instructions. To examine pathology changes, the lung tissues were fixed for H&E staining according to a standard protocol [25].

### Statistical analysis

All experiments were repeated at least thrice with three to six replicates. Data were expressed as mean ± standard deviation (SD) from at least three replicates. Data analysis was performed by two-tailed Student’s t-test. The difference was regarded as statistical significance if *p* < 0.05.

## Results

### Toxicity assessments of ENMs in G^+^ and G^−^ bacteria

**Fig. 1:**
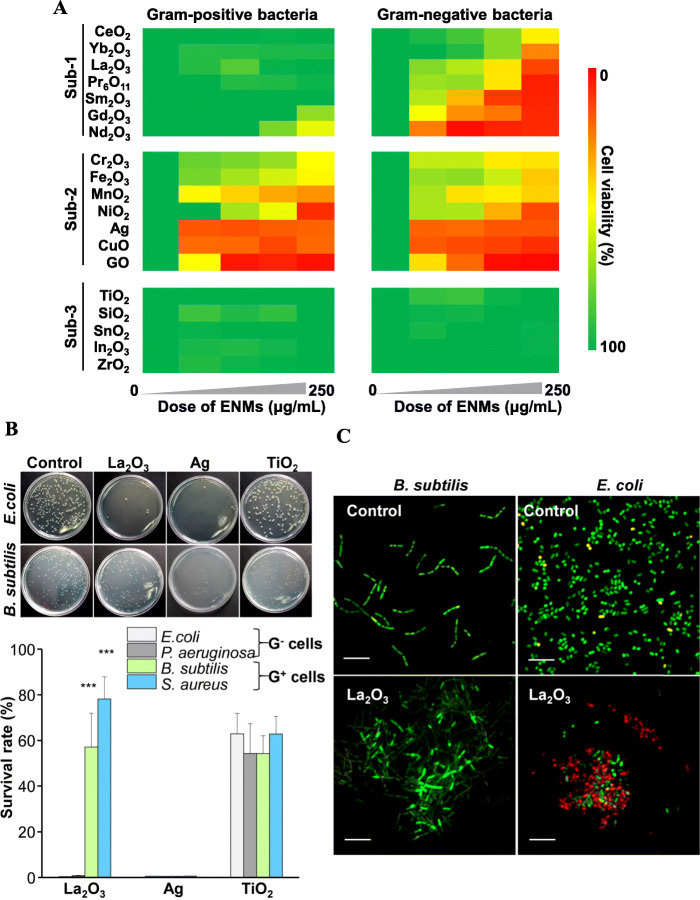
Comparison of cell viabilities of G^+^ and G^−^ bacteria exposed to ENMs. **a** Heat maps displaying the toxicity of ENMs in *E. coli* and *B. subtilis* cells. *E. coli* and *B. subtilis* suspensions were exposed to different nanoparticles for 2 h. Then the cell viabilities were determined by bacteria counting colorimetric assay. Three replicates were conducted for each dosage. **b** Survival rate examination by CFU assay. The colonies of *E. coli*, *P. aeruginosa*, *S. aureus* and *B. subtilis* were counted after exposure to La_2_O_3_ at 250 μg/mL. Columns and error bars represent the mean and the standard deviation of three replicates. ***p < 0.001 compared to G^−^ cells by two-tailed Student’s t-test. **c** Live/Dead staining. *E. coli* and *B. subtilis* cells were incubated with La_2_O_3_ and stained by a Live/Dead staining kit for confocal microscopy imaging. Scale bar is 7 μm

Nineteen ENMs including REOs, metal-based and carbon-based nanoparticles were characterized by zeta potential and hydrodynamic analyzer. As shown in Table S1, all of the ENMs except for graphene oxide (GO) exhibited spherical morphologies with primary sizes at 10 ~ 100 nm and significant agglomerations with hydrodynamic sizes at 70 ~ 460 nm. Their zeta potential ranged from − 23.7 to 36.4 mV. In terms of the tested bacteria strains, they should meet the following criteria: i) reported in pulmonary microflora; ii) commercially available; iii) biosafety level ≤ BSL2. As a result, four G^−^ strains (*E. coli*, *P. aeruginosa, V. harveyi, S. typhimurium*) and two G^+^ strains (*S. aureus, B. subtilis*) were selected to examine the toxic effects of ENMs (Table S2). To assess the hazard effects of ENMs on G^+^ and G^−^ bacteria, cell viabilities of *E.coli* and *B. subtilis* were examined by colorimetric assay after exposure to ENMs at 0–250 μg/mL. The acquired cell viability data were integrated into a heat map to visualize the toxic effects on *E.coli* and *B. subtilis* cells, where red, yellow and green colors represented strong, moderate and negligible toxicity effects. As shown in Fig. 1a, the ENMs could be divided into three subgroups (sub-1 to 3) based on their effects on two bacterial strains. While sub-3 group, including TiO_2_, SiO_2_, SnO_2_, In_2_O_3_ and ZrO_2_ nanoparticles showed green dots with > 82.0% cell viabilities on both *E. coli* and *B. subtilis*, sub-2 group involving Cr_2_O_3_, Fe_2_O_3_, MnO_2_, NiO_2_, Ag, CuO and GO showed from green to red with the exposure dosageincrement. By comparison, all seven REOs (sub-1 group) including CeO_2_, Yb_2_O_3_, Pr_6_O_11_, Sm_2_O_3_, La_2_O_3_, Gd_2_O_3_ and NdO_2_ displayed significant decrement of cell viability in *E. coli*, but limited decline in *B. subtilis*. Moreover, La_2_O_3_, Ag and TiO_2_ were selected as representative particles of three subgroups of ENMs to detect their effects in *E.coli*, *P. aeruginosa*, *S. aureus* and *B. subtilis* by CFU assays (Fig. 1b). As a result, Ag and TiO_2_ nanoparticles exhibited either strong toxicity (survival rates < 0.01%) or negligible bactericidal effects in G^−^ and G^+^ bacteria. In contrast, La_2_O_3_ treatment led to < 0.4% survival rates in two G^−^ bacterial strains, but limited effects in G^+^ bacteria. Moreover, La_2_O_3_ showed dose-dependent toxicity in other two G^−^ strains, including *V. harveyi* and *S. typhimurium* (Figure S1). To further visualize the impact of La_2_O_3_ in G^−^ and G^+^ bacteria, we conducted a confocal microscope following by the Live/Dead staining. As shown in Fig. 1c, a majority of *E. coli* cells exposed to La_2_O_3_ allowed PI across membranes to bind with DNA molecules and displayed intense red fluorescence, while most *B. subtilis* cells emitted green fluorescence by SYTO9.

### Biotransformation of La_2_O_3_ at the nano-bio interfaces

**Fig. 2:**
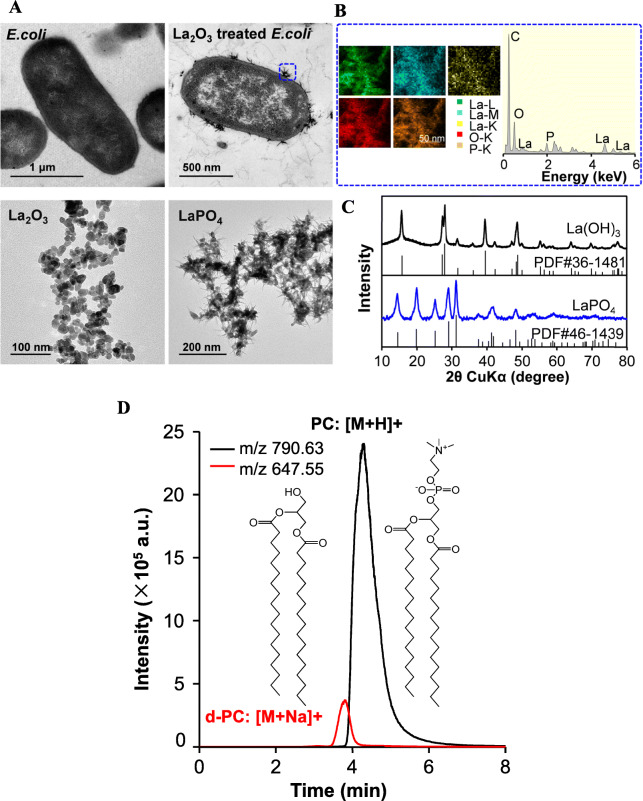
Assessments of the interaction at La_2_O_3_-membrane interface. **a** Visualizing La_2_O_3_ in *E. coli* by TEM. *E. coli* cells were exposed to La_2_O_3_ at 250 μg/mL. After fixation in glutaraldehyde, cells were stained and sliced for TEM observation. Pristine La_2_O_3_ and LaPO_4_ were also examined by TEM. **b** EDX spectra and elemental mapping analysis. The chemical composite of pellets transformed at the interfaces of cell membrane were examined. **c** XRD analysis for the pristine particles and LaPO_4_. **d** LC-MS analysis of PC exposed to La_2_O_3_. After 4 h incubation with La_2_O_3_, PC was collected and extracted for LC-MS examination

TEM was performed to examine the interaction of La_2_O_3_ and bacterial cells. As shown in Fig. 2a, spherical La_2_O_3_ nanoparticles bound with *E. coli* cells and transformed to needle-like pellets intercalated into cell membranes. These transformed pellets containing abundant phosphorous elements were distinctively different to the pristine La_2_O_3_ nanoparticles, but similar to hexagonal LaPO_4_, evidenced by EDX analysis and XRD assessment (Fig. 2b-c). To examine the transformation at the nano-bio interfaces, phosphatidylcholine (PC) liposomes were prepared to simulate the phospholipid outer layer of G^−^ bacteria and subjected to analysis by LC-MS after incubation with La_2_O_3_. As a result, beside of the PC molecular ion peak at m/z 790.63, a dephosphorylation peak (m/z 647.55) was examined, yielding 8.3% dephosphorylated PC (de-PC) molecules (Fig. 2d). In addition, disaccharide with β-1,4 glycosidic bonds was used to simulate peptidoglycan of G^+^ bacteria. LC-MS analysis showed that La_2_O_3_ had no influence on the chemical structure of disaccharide molecules (Figure S2).

### Molecular dynamics simulation of membrane damages

**Fig. 3:**
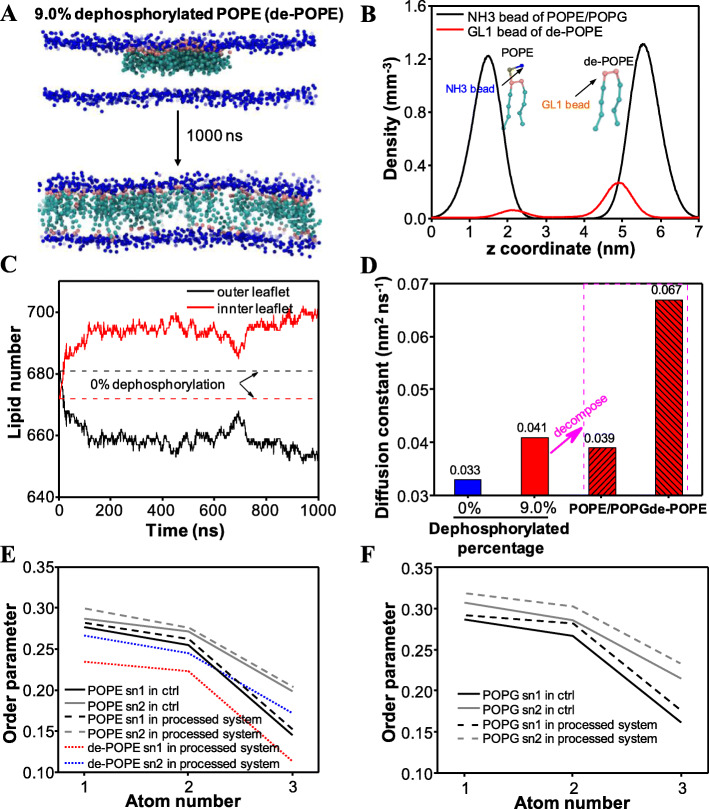
Molecular dynamics simulation revealing the robust disruption of cell membranes triggered by dephosphorylated lipids. **a** Structural information of the processed membrane with 9.0% de-POPE. Blue dots represent heads of normal POPE and POPG while other dots indicate de-POPEs. The upper and lower pictures are the initial and final configurations of membrane for 1000 ns simulation. **b** Densities of NH3 beads of POPE/POPG (black) and GL1 beads of de-POPE (red) along z axis. The inserted pictures are the representations of NH3 bead of POPE/POPG and GL1 bead of de-POPE. **c** Lipid numbers of two membrane leaflets in control and processed systems. The dashed lines indicate the lipid numbers of the normal membrane, whereas the solid lines indicate the lipid numbers of the processed membrane (dephosphorylated membrane). **d** Lateral diffusion constants of different lipids. The left two histograms exhibit the lateral diffusion constants of the entire membrane in control (blue) and processed (red) systems. The right two histograms decompose the diffusion constant of the processed membrane into the diffusion constants of normal POPE/POPG and de-POPE. **e** Order parameters of POPE in control system and POPE/de-POPE in processed system. sn-1 and sn-2 indicate two tails of each lipid. **f** Order parameters of POPG in control and processed systems

MD simulations were performed to analyze the impact of dephosphorylation on the behaviors of cell membrane at the molecular level. We simulated two systems: one for normal bacterial membrane (served as the control system) and one for dephosphorylated membrane. The normal bacterial membrane consists of an outer leaflet with pure palmitoyloleoylphosphatidylethanolamine (POPE) and an inner leaflet with mixed POPE/palmitoyloleoylphosphatidylglycerol (POPG) of 3:1 proportion. Meanwhile, the dephosphorylated membrane was prepared via partial dephosphorylation of normal membrane lipids (Figure S3). The processed membrane with a dephosphorylated proportion of 9.0% (Fig. 3a) was modeled based on the experimental findings as discussed above. After an unbiased simulation for the duration of 1000 ns, the dephosphorylated membrane showed an obviously morphological alteration as compared with the initial setup (Fig. 3a). Specifically, the dephosphorylated POPEs (de-POPEs) dispersed freely inside the two-dimensional bilayer. To quantify the distribution of different lipids, we calculated the density distributions of POPE/POPG heads and de-POPE heads along the normal direction after 1000 ns. As shown in Fig. 3b, de-POPEs were detected in either outer leaflet or inner leaflet. Lipid number alterations also demonstrated the same tendencies (Fig. 3c). By contrast, in the normal membrane, the lipid numbers in two leaflets presented two constant values. Subsequently, we computed the membrane fluidity by computing the lateral diffusion constant. As shown in Fig. 3d, the lateral diffusion constant of membrane in the processed system (9.0% dephosphorylation) was 0.041, exhibiting higher than that in the control system. By decomposing the lateral diffusion constant into the lateral diffusion constants of normal lipids and de-POPEs, we found that de-POPEs presented a very large value of lateral diffusion constant, whereas the diffusion constant change of POPE/POPG was much milder. Moreover, the order parameters of all lipids in two systems were assessed (Fig. 3e-f). Notably, the order parameters of two tails (i.e., sn-1 and sn-2) of both POPE and POPG in processed system became larger than that in the control system. In contrast, the order parameters of de-POPE decreased dramatically.

### Assessment of membrane disruption

**Fig. 4:**
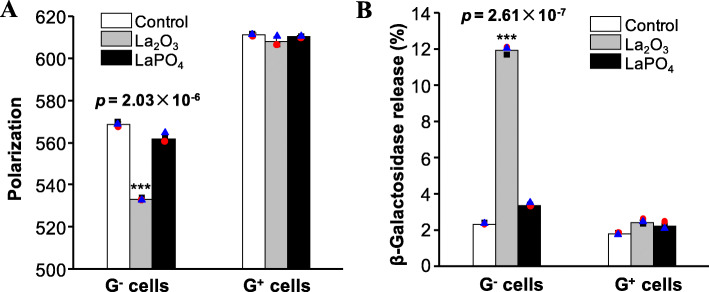
Validation of La_2_O_3_ induced membrane fluidity and permeability changes. **a** Detection of the polarization of TMA-DPH intercalating in cell membranes. *E. coli* and *B. subtilis* cells were incubated with 250 μg/mL La_2_O_3_ or LaPO_4_ for 2 h. TMA-DPH probe (5 μL, 2 mM) was added to intercalate bacterial membrane, and the polarization of TMA-DPH was measured at ex/em 360 nm/460 nm by a 66 FP module at Microplate Reader. **b** Examination of β-galactosidase release. After treatment, the supernatants of particle treated cells were collected to incubate with β-galactosidase substrate for luminance examination. Columns and error bars represent the mean and the standard deviation of three replicates. ***p < 0.001 compared to controls by two-tailed Student’s t-test

To verify the effect of La_2_O_3_ on phospholipid lateral and vertical diffusion by MD simulations, we monitored membrane fluidity and permeability in G^−^ and G^+^ cells by fluorescence polarization and β-galactosidase release assays, respectively. As shown in Fig. 4a, *E. coli* cells exposed to La_2_O_3_ showed a fluorescence polarization (FP) value of 533.3 ± 0.6, significantly lower (*p* = 2.03 × 10^− 6^) than the control cells. Interestingly, *E. coli* cells exposed to LaPO_4_ showed 562.0 ± 2.0 FP value, similar to the control cells. By comparison, in *B. subtilis* cells, both La_2_O_3_ and LaPO_4_ treatments exhibited the approximate FP values with the control group. To assess the impacts of La_2_O_3_ on membrane permeability, β-galactosidase release was detected in G^−^ and G^+^ cells by a luminescent substrate [10]. As shown in Fig. 4b, La_2_O_3_ nanoparticles induced 12% β-galactosidase release from *E. coli*, significant higher (*p* = 2.61 × 10^− 7^) than the control and LaPO_4_ treated cells, whereas La_2_O_3_ treatment elicited limited β-galactosidase release in *B. subtilis* cells.

### Effects of La_2_O_3_ on microbial communities

**Fig. 5:**
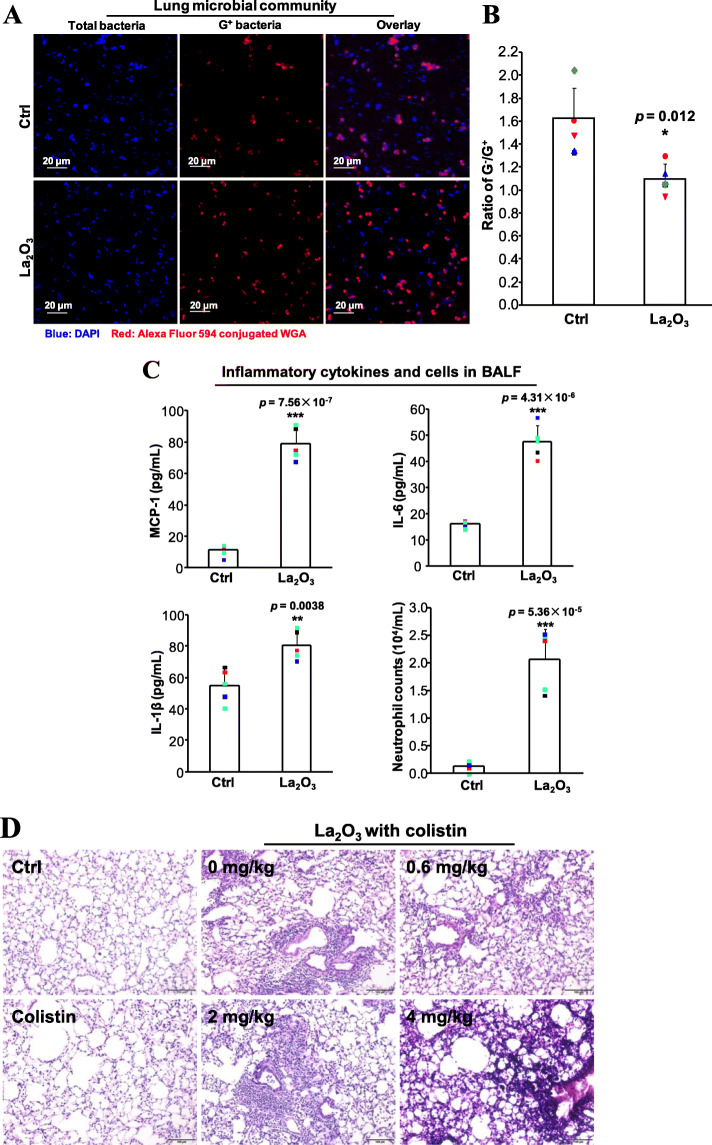
Evaluation of the disruption of La_2_O_3_ in lung. **a** The images of lung microbial communities. Animals were exposed to 2 mg/Kg La_2_O_3_ by oropharyngeal aspiration for 24 h (*n* = 5). Lung microbes were collected in BALF by centrifugation. The collected bacteria were subjected to confocal microscopy imaging after DAPI and Alex Flour 549 conjugated WGA staining. **b** The ratios of G^+^/G^−^. The percentage of G^+^ and G^−^ cells were calculated based on the confocal images. The ratios were presented as mean values ± SD from five mice. *p < 0.05 compared to control group by two-tailed Student’s t-test. **c** Pro-inflammatory cytokine production and neutrophil counts in BALF. Immune cells in BALF were concentrated on glass slides by cytospin, fixed and stained by Quick-Diff for cell counting. Pro-inflammatory cytokines were determined by ELISA. Cell counting and cytokine data are presented as mean values ± SD derived from five mice. **p < 0.01 and ***p < 0.001 compared to controls by two-tailed Student’s t-test. **d** H&E staining images of lung tissues exposed to La_2_O_3_ with different concentrations of colistin. Mice exposed to saline or colistin were used as control groups. Scale bars represent 100 μm

To evaluate the effect of La_2_O_3_ on microbial communities, bacterial gram staining was conducted to examine the ratio of G^−^ and G^+^ strains in lung microbial communities. G^+^ strains in lung samples could be positively stained by Alexa Fluor 594 labeled wheat germ agglutinin (WGA), emitting red fluorescence under confocal microscope. The impacts of La_2_O_3_ on each microbial community were determined by Image J analysis of the portion of G^+^ cells. As shown in Fig. 5a, while a considerable portion (60.8%) of G^−^ cells denying the binding to WGA could be visualized in untreated lung microbial communities, La_2_O_3_ treatment showed substantial red fluorescent G^+^ cells and relatively lower abundance of G^−^ cells (51.3%), resulting in the decrease of G^−^/G^+^ ratios from 1.56 to 1.10 (Fig. 5b). This microbial community changes were accompanied by significant increments of neutrophil count and cytokines (IL-6, IL-1β and MCP-1) in bronchoalveolar lavage fluid (BALF) (Fig. 5c). To explore the relationships between pulmonary microflora imbalance and inflammation, we deliberately disrupted the microbial communities in mouse lungs by exposure to different concentrations of colistin, which has little effect in G^+^ bacteria but specifically kills G^−^ bacteria. These microflora-disrupted mice were exposed to La_2_O_3_. As expected, dramatic reductions of G^−^/G^+^ ratios (1.10 to 0.51) were detected in BALF of colistin-treated mice (Figure S4). Notably, the pro-inflammatory cytokines (IL-6, IL-1β and MCP-1) as well as neutrophil counts were significantly increased along with the reduction of G^−^/G^+^ ratios (Table 1). Both IL-6 and neutrophil cells showed strong correlations with G^−^/G^+^ ratios, evidenced by their correlation coefficient |r| > 0.8. Consistently, G^−^/G^+^ ratio dependent focal inflammation could be visualized around the airways of lungs exposed to La_2_O_3_ (Fig. 5d).

**Table 1:** Biological response in BALF after La_2_O_3_ and colistin exposure

Ratio of G^**−**^/G^**+**^	Inflammatory cytokines (pg/mL)			Cells (10^**4**^)
	IL-1β	MCP-1	IL-6	Neutrophils
R1 = 1.10	80.6 ± 9.3	78.9 ± 10.3	47.5 ± 6.2	2.1 ± 0.6
R2 = 0.74	79.8 ± 13.4	78.0 ± 15.1	66.2 ± 13.6	6.7 ± 1.9
R3 = 0.63	80.0 ± 17.3	80.7 ± 15.5	108.1 ± 15.7	7.8 ± 2.3
R4 = 0.51	144.9 ± 32.2	135.4 ± 24.5	196.7 ± 24.2	14.6 ± 2.3
Pearson’s correlation |r|	0.61	0.63	0.83	0.92

Besides that, the effects of La_2_O_3_ exposure to environmental microbiology were examined by Gram staining and 16S rRNA sequencing. As shown in Figure S5, the ratio of G^−^/G^+^ in soil microbial communities decreased from 1.96 to 1.07 after La_2_O_3_ exposure. To visualize the detailed microbial community changes induced by La_2_O_3_, the relative abundance values (Z scores) of top 35 genera identified by 16S rRNA-based sequencing were integrated into a heat map. As the visual data displayed, more than ten G^−^ strains showed lower abundance in La_2_O_3_-treated soil microbial community (Figure S6A). Consistently, a significant decrement of G^−^/G^+^ ratios could be detected in the soil microbial community after La_2_O_3_ exposure (Figure S6B).

## Discussion

### Biotransformation of REOs elicited disruption of G^−^ cell membrane

Since MIEs play decisive role in adverse outcome pathways [26], we speculated that the MIEs at the nano-bio interfaces may be responsible for the distinct bactericidal effects of La_2_O_3_. Interestingly, La_2_O_3_ was found to associate with the membranes of *E. coli* cells and transform into needle-like LaPO_4_ precipitates. This is because lanthanides have strong binding affinity (Ksp ~ 10^–25.6^) [27] with phosphate to form hexagonal structures. La ions might bind with two types of phosphates, including free phosphates in biosystem and conjugated phosphate moieties with biomolecules (phospholipids, phosphorylated proteins, *etc*) of membranes. We speculated that dephosphorylation of membrane biomolecules may be responsible for the bactericidal effects of La_2_O_3_. The dephosphorylated PC molecules and unchanged disaccharide molecules detected by mass spectrometry strongly suggested that the dephosphorylation of phospholipids on G^−^ cell membranes might be the key MIE responsible for the bactericidal effects of rare earth nanoparticles.

MD simulation disclosed that La_2_O_3_-induced dephosphorylation resulted in bacterial membrane disruption with a much less ordered configuration, and thus increased the lateral and vertical diffusions of phospholipids. The results of β-galactosidase release revealed that La_2_O_3_ could induce an increment of membrane permeability, which was consistent with the MD result of increased lateral diffusions of phospholipids. Additionally, a fluorescent TMA-DPH probe capable of localizing membrane interiors for assessment of bacterial membrane fluidity in situ was exploited to testify the vertical diffusions of phospholipids. Specifically, La_2_O_3_ treatments induced a significant reduction of FP value in G^−^ cells compared to G^+^ cells, demonstrating an increased membrane fluidity of G^−^ cell membrane. All considered, the disruption of membranes by La_2_O_3_ in G^−^ bacterial cells likely involved four sequential processes: i) transformation of La_2_O_3_ into LaPO_4_; ii) dephosphorylation of phospholipids in G^−^ cell membranes; iii) induction of an unordered configuration of phospholipids; iv) increment of both membrane fluidity and permeability.

### REOs causing microbiota imbalance

Recent studies showed that bactericidal effects of REOs could be attributed to their impacts on biological media surrounding cells, ROS generation and metal ion release. For instance, La_2_O_3_ nanoparticles were found to scavenge phosphate from media to inhibit the growth of *Escherichia coli, Staphylococcus carnosus, Penicillium roqueforti* and *Chlorella vulgaris* [14]. The potential toxicity of CeO_2_ was ascribed to the ROS generation from the fast valence exchange between Ce(III) and Ce(IV) [28]. Additionally, ionic release from REO nanoparticles was also found to impact the viability of *E. coli* [29]. Although the toxic effects of REOs have been identified in a few bacteria strains, no attempts were made to differentiate the effects of REOs on G^−^ and G^+^ cells. Our study disclosed the mechanisms of La_2_O_3_-elicited imbalance of the lung and soil microbial communities. Moreover, severe pulmonary inflammatory responses were examined in La_2_O_3_ treatment by oropharyngeal aspiration, evidenced by the increments of neutrophil counts and cytokine release in BALF as well as focal inflammation in lung sections. These pathology changes may further lead to pneumoconiosis extensively reported in RE miners [30]. In terms of the toxicology mechanisms involved in rare earth pneumoconiosis, we have demonstrated a NLRP3 inflammasome pathway in macrophage-like cells responsible for REO-induced lung fibrosis by eliciting lysosomal damages, cathepsin B release, and IL-1β release [31]. While microbial communities have been recently found to play a major role in many diseases, including tumors [32], metabolic diseases [33], neurodegenerative disorders and inflammatory bowel diseases [34, 35], few studies explore the relationships between lung microflora imbalance and pulmonary diseases. In our study, lung toxicity results implied that microflora might play a protective role in La_2_O_3_-induced pulmonary inflammation. Since similar biotransformation behaviors have been identified in more REOs, such as Gd_2_O_3_, Nd_2_O_3_, Sm_2_O_3_, and Yb_2_O_3_ nanoparticles [31], these particles are likely to induce similar microbiota imbalance effects to La_2_O_3_.Moreover, we speculated that the impacts of microflora imbalance on pulmonary hazard effects induced by other stimuli (e.g.*,* virus, air pollutants and fine particulates) may deserve more explorations.

### Relevance of REO doses and exposure routes in real scenarios

Rare earth elements have wide applications in magnets, catalysts, metal alloys, electronics, glass, ceramics and new materials. There might be two major sources of nano-sized rare earth particles, including engineered rare earth nanoparticles and ultrafine particulates in RE industries. During the life cycle of rare earth nanoproducts, the particles have exposure risks to humans by inhalation, ingestion, dermal exposure and intravenous injection [7]. In addition, fine particulates from industries may be released into water, soil or air in RE mining, polishing and waste treatments. According to a Health Hazard Evaluation (HHE) report (No. HETA-88-0166-1944), OSHA’s permissible exposure limit (PEL) for RE (e.g., Y, Nd and Dy) is 1 mg/m^3^. Based on a dose calculation equation for inhaled particulates (Formula S1 in Supplemental materials) [7, 36], a worker exposure to 1 mg/m^3^ respirable dust for 8 h/day over a five-month time period over a 10-year working time could lead to a lung burden of 2.2 mg/m^2^, which is comparable to 4.4 mg/Kg NPs in mice. Assuming a homogeneous distribution of NP in alveoli, the corresponding in vitro NP dose would be 0–170 μg/mL. In this study, the in vitro and in vivo doses of tested materials are at 0–250 μg/mL and 2 mg/Kg, respectively. These doses are relevant to the possible occupational exposure in real scenarios.. In soil samples, the average concentrations of RE elements were reported to be 180 mg/Kg in China [37]. In our studies, La_2_O_3_ at 200 μg/mL was exposed to microbe communities extracted from 0.005 Kg soils. It was equivalent to 200 mg/Kg RE nanoparticles in soil. Overall, the dosages of La_2_O_3_ tested in this study were relevant to real scenarios of occupational exposure in miners or soils close to RE mine. Due to the complexity and low abundance of pulmonary microflora, it’s difficult to validate the bactericidal mechanism in animals. This challenge necessitated the exploration of culturomics for pulmonary microbes, including ex vivo high-throughput culture methods of pulmonary bacteria and high-sensitive analytical methods to identify low abundant microbes from a complex population. In terms of real respiratory scenarios, aerosol inhalation exposure is more relevant. The results acquired in this study may need further validations.

## Conclusion

In this study, La_2_O_3_ was found to show significant toxicities on G^−^ bacteria, but limited effects on G^+^ cells. Biotransformation of La_2_O_3_ into LaPO_4_ at nano-bio interface elicited dephosphorylation of membrane phospholipids, a much less ordered configuration of phospholipids and increments of membrane fluidity and permeability. In real exposure scenarios, La_2_O_3_ was demonstrated to disrupt the balance of G^−^ and G^+^ bacteria in lung and soil microbial communities. Notably, we disclosed a strong correlation between microflora imbalance and lung inflammation in mouse lungs received La_2_O_3_ exposure. Overall, our study will provide a clearer and more comprehensive understanding of the hazard effects induced by REOs.

## Supplementary Information

**Additional file 1.**

## Data Availability

All data displaying in this paper are available in a generalist repository, Harvard Dataverse, by following URL: https://dataverse.harvard.edu/dataset.xhtml?persistentId=doi:10.7910/DVN/2DG4YP
