# Supplementary material for: Biotransformation of rare earth oxide nanoparticles eliciting microbiota imbalance
Source: Part Fibre Toxicol. 2021 Apr 26;18:17. doi: 10.1186/s12989-021-00410-5 (PMC8077720; doi:10.1186/s12989-021-00410-5)
Supplement: Supplementary file 1 — Additional file 1. [file 12989_2021_410_MOESM1_ESM.docx]

Supplementary Information for

**Biotransformation of Rare Earth Oxide Nanoparticles Eliciting Microbiota Imbalance**

Huizhen Zheng^1#^, Zonglin Gu^2#^, Yanxia Pan^1^, Jie Chen^1^, Qianqian Xie^1^, Shujuan Xu^1^, Meng Gao^1^, Xiaoming Cai^3^, Shengtang Liu^1^, Weili Wang^1^, Wei Li^1^, Xi Liu^1^, Zaixing Yang^1*^, Ruhong Zhou^1,4^, Ruibin Li^1*^

^1^State Key Laboratory of Radiation Medicine and Protection, School for Radiological and Interdisciplinary Sciences (RAD-X), Collaborative Innovation Center of Radiological Medicine of Jiangsu Higher Education Institutions, Soochow University, Suzhou, 215123, Jiangsu, China;

^2^Institute of Quantitative Biology, Department of Physics, Zhejiang University, Hangzhou 310027, Zhejiang, China;

^3^School of Public Health, Jiangsu Key Laboratory of Preventive and Translational Medicine for Geriatric Diseases, Soochow University, Suzhou, 215123, Jiangsu, China;

^4^Department of Chemistry, Columbia University, New York, NY 10027, USA

^#^ Equal contributions

* Correspondence to

Dr. Ruibin Li Email: [liruibin@suda.edu.cn](mailto:liruibin@suda.edu.cn)

Tel: +86-512-65880062

Dr. Zaixing Yang Email: [zxyang@suda.edu.cn](mailto:zxyang@suda.edu.cn)

Tel: +86-512-65882425

**Calculation of nanoparticle inhalation exposure doses**

In the animal experiments, the dosage of La_2_O_3_ has been reference from the occupational environment and the lung inhalation concentrations. In detail, it was calculated by the following formula[[1](#_ENREF_1)]:

$DOA=\frac{C_{0}\times IR\times T}{\mathrm{TA}_{\mathrm{human}}}\times\frac{\mathrm{TA}_{\mathrm{mice}}}{\mathrm{BW}}$ (S1)

where DOA is the exposure dose of animals (mg/Kg); C_0_ is the level of NPs in air (mg/m^3^); IR is the intake rate of a worker (10 m^3^/24 h); TA is the tissue area (TA_human_ = 75 m^2^, TA_mice_ = 0.05 m^2^); T is the working time (h).

**Extraction of** **soil microbial communities**

To assess the effects of La_2_O_3_ nanoparticles on soil microbial communities, the soil samples were collected. In detail, the soil samples were collected in sterile tubes from the surface horizon (0-10 cm) under a tree at lakeside in Soochow University, China. The soil samples were transported by ice boxes to the laboratory and sieved by gauze to remove the stone and plant residues. Then, the soil suspensions were obtained by weighing 10 g sieved soil into a 250 mL conical flask containing 100 mL saline. After thoroughly mixing in a shaker at 200 rpm for 1 h, the soil suspensions were centrifuged at 200 g for 5 min to remove insoluble granules. The supernatant was collected and subsequently centrifuged at 6000 g for 5 min to separate the soil bacterial cells. Three independent soil samples were obtained.

After washing with saline for five times to discard the latent phosphate groups, each soil sample was dispersed in 10 mL saline and partitioned into two subgroups, respectively. One was treated with 200 μg/mL La_2_O_3_ nanoparticles, and the other was served as control without treatment. After 4 h incubation, the bacterial cells were collected by 6000 g centrifugation for 5 min. Furthermore, total nucleic acids of all tested samples were extracted using cetyltrimethylammonium bromide (CTAB) method[[2](#_ENREF_2)]. The qualities of genomic DNA were detected by gel electrophoresis and NanoDrop 1000 (Thermo Scientific, USA)[[3](#_ENREF_3)]. The extracted total DNA was stored at -80 °C until further processing.

**16S rRNA gene sequencing**

16S rRNA genes in distinct regions (16S V4) of extracted bacterial DNA were amplified using specific primer (515F-806R) in 200 µL PCR tubes with Mastercycler® nexus (Eppendorf, USA). In details, all PCR reactions were carried out with 15 µL of Phusion® High-Fidelity PCR Master Mix (New England Biolabs), 2 µL primers (0.2 µM), and about 10 ng template DNA with DI water up to 50 µL. Thermal cycling process consisted of initial denaturation at 98 ℃ for 1 min, 30 cycles of PCR reactions at 98 ℃ for 10 s, annealing at 50℃ for 30 s, and elongation at 72 ℃ for 30 s, followed by 5 min at 72 °C as final extension. Then, the PCR products were mixed with the same volume of 1X loading buffer to operate electrophoresis on 2% agarose gel. The target band was purified with Qiagen Gel Extraction Kit (Qiagen, Germany). TruSeq® DNA PCR-Free Sample Preparation Kit (Illumina, USA) was added to each purified PCR product to generate sequencing libraries according to the manufacturer's recommendations. The library quality was checked on the Qubit@2.0 Fluorometer (Thermo Scientific, USA) and Agilent Bioanalyzer 2100 system. Finally, the library was sequenced on an Illumina NovaSeq platform and 250 bp paired-end reads were generated.

Organisms were identified by comparing consensus sequences to a database library of known 16S rRNA gene sequences in GenBank (http://www.ncbi. nlm.nih.gov/blast/Blast.cgi) by multiple sequence alignment. We performed Silva Database (<http://www.arb-silva.de/>) to annotate taxonomic information based on Mothur algorithm. Standardized relative abundance values (Z values) were normalized by taxa relative abundance data using the Z score method, which were defined by the following formula:

$Z =(x-\mu)/\sigma$ (S2)

Where x is the raw score and represents the abundance of one operational taxonomic unit (OTU) in one sample; μ is the population mean and represents the mean abundance for that OTU; σ is the population standard deviation and represents the standard deviation of that OTU.

**Table S1.** **Sources and properties of ENMs**

| **ENMs** | **Vendor** | **Primary size**  **(nm)** | **Hydrodynamic size (nm)** | **Zeta potential (mV)** |
| --- | --- | --- | --- | --- |
| La_2_O_3_ | NanoAmor | 15-30 | 460.0 ± 45.0 | 23.0 |
| Pr_6_O_11_ | NanoAmor | 15-30 | 310.8 ± 10.3 | 13.8 |
| Y_2_O_3_ | NanoAmor | 32-36 | 289.8 ± 20.3 | 24.3 |
| Sm_2_O_3_ | NanoAmor | 15-30 | 340.8 ± 11.3 | 25.0 |
| CeO_2_ | NanoAmor | 15-30 | 282.6 ± 30.3 | 13.7 |
| Yb_2_O_3_ | NanoAmor | 10-80 | 380.2 ± 25.3 | 15.5 |
| Nd_2_O_3_ | NanoAmor | 15-30 | 322.8 ± 19.3 | 36.4 |
| Gd_2_O_3_ | NanoAmor | 15-30 | 313.1 ± 13.2 | 18.1 |
| Cr_2_O_3_ | US Nano | 60 | 349.6 ± 15.3 | 4.4 |
| Fe_2_O_3_ | US Nano | 30 | 70.1 ± 19.3 | -2.1 |
| MnO_2_ | US Nano | 100 | 323.4 ± 4.2 | -23.4 |
| Ni_2_O_3_ | US Nano | 80 | 369.1 ± 36.2 | 28.6 |
| Ag | UIV Chem | 25-50 | 156.6 ± 10.1 | -4.5 |
| CuO | NanoAmor | 15-30 | 490.6 ± 40.9 | 4.6 |
| GO | In our lab[[4](#_ENREF_4)] | 100-500 | 286.8 ± 18.8 | -24 |
| TiO_2_ | US Nano | 30 | 146.0 ± 16.2 | -30.5 |
| SiO_2_ | NanoAmor | 20-30 | 110.2 ± 2.6 | -23.7 |
| SnO_2_ | US Nano | 35-55 | 420.0 ± 5.6 | 21.8 |
| In_2_O_3_ | NanoAmor | 20-70 | 186.7 ± 3.9 | 28.1 |
| ZrO_2_ | US Nano | 30-50 | 412.1 ± 9.4 | -11.9 |

**Table S2. Tested bacterial strains**

|  | Strains | ATCC number |
| --- | --- | --- |
| G^-^ bacteria | *Escherichia coli* | MG1655 |
|  | *Pseudomonas aeruginosa* | BAA-47 |
|  | *Vibrio harveyi* | BB170 |
|  | *Salmonella typhimurium* | 14028 |
| G^+^ bacteria | *Bacillus subtilis* | 6633 |
|  | *Staphylococcus aureus* | 6538 |

**Figure S1. The effect of La_2_O_3_ on bacterial cell viability**

Two G^-^ stains including *V. harveyi* and *S. typhimurium*, were treated with La_2_O_3_ suspensions at 0-250 μg/mL for 2 h. Bacterial viability were examined by a bacterial counting colorimetric assay kit. Three replicates were performed for each dosage.

**Figure S2. LC-MS analysis of cellobiose**

Cellobiose was dissolved in DI H_2_O at 1 mg/mL and incubated with 250 μg/mL La_2_O_3_ particles at 37 ℃ for 4 h. Then cellobiose was collected for LC-MS examination (right). The untreated cellobiose was used as control (left).

**Figure S3.** **Design of dephosphorylated POPE based on normal POPE**

The dephosphorylated POPE is designed by deleting the two head beads of normal POPE.

**Figure S4. Assessment of the lung microbial communities by Gram staining**

Animals were exposed to 2 mg/Kg La_2_O_3_ with different concentration of colistin by oropharyngeal aspiration for 24 h (n = 5 mice). The lung microbial communities in BALF were collected by centrifugation and stained by DAPI and Alex Flour 549 conjugated WGA for 15 min. The total and G^+^ cells were discriminated by blue and red fluorescence under confocal microscopy. Scale bars represent 25 μm.

**A B**

**Figure S5. Assessment of the soil microbial communities by Gram staining**

**A)** Confocal imaging of G^+^ bacteria in soil microbial communities. Microbial communities extracted from soil were incubated with 200 μg/mL La_2_O_3_ in saline for 4 h. The treated cells were stained by DAPI and Alex Flour 549 conjugated WGA for 15 min, followed by confocal microscopy imaging. Scale bars represent 25 μm. **B)** The ratios of G^+^/G^-^. The percentage of G^+^ and G^-^ cells were calculated by Image J based on the confocal images. Data are represented as mean values ± SD, which were obtained by three independent experiments. **p* < 0.05 compared to control group by two-tailed Student t-test.

**A B**

**Figure S6. Comparison of soil microbial communities before and after La_2_O_3_ treatment by 16S rRNA sequencing**

A) A heat map displaying Z values of the top 35 genera. B) Relative abundances of genera clustered into G^+^ and G^-^ strains. Soil microbial samples were treated by 200 μg/mL La_2_O_3_ for 4 h. 16S rRNA sequencing was performed on the extracted DNA from soil microbial samples by an Illumina NovaSeq platform. Z values in the heat map represented the relative abundance of individual genera were calculated by equation 1 in Supplemental materials. The sequencing experiments were performed with three independent soil samples.

**References**

[1] Cai X, Liu X, Jiang J, Gao M, Wang W, Zheng H, et al. Molecular mechanisms, characterization methods, and utilities of nanoparticle biotransformation in nanosafety assessments. Small. 2020; 1907663.

[2] Guerra V, Beule L, Lehtsaar E, Liao HL, Karlovsky P. Improved protocol for DNA extraction from subsoils using phosphate lysis buffer. Microorganisms. 2020; 8: 532.

[3] Pereira JC, Chaves R, Bastos E, Leitão A, Guedes-Pinto H. An efficient method for genomic DNA extraction from different molluscs species. Int J Mol Sci. 2011; 12: 8086-95.

[4] Li R, Mansukhani ND, Guiney LM, Ji Z, Zhao Y, Chang CH, et al. Identification and optimization of carbon radicals on hydrated graphene oxide for ubiquitous antibacterial coatings. ACS Nano. 2016; 10: 10966-80.
